# Epstein–Barr Virus BALF0 and BALF1 Modulate Autophagy

**DOI:** 10.3390/v11121099

**Published:** 2019-11-27

**Authors:** Zhouwulin Shao, Chloé Borde, Frédérique Quignon, Alexandre Escargueil, Vincent Maréchal

**Affiliations:** Sorbonne Université, INSERM, Centre de Recherche Saint-Antoine, F-75012 Paris, France

**Keywords:** Epstein–Barr virus, BALF0/1, BALF1, BALF0, autophagy, vBcl-2

## Abstract

Autophagy is an essential catabolic process that degrades cytoplasmic components within the lysosome, therefore ensuring cell survival and homeostasis. A growing number of viruses, including members of the Herpesviridae family, have been shown to manipulate autophagy to facilitate their persistence or optimize their replication. Previous works showed that the Epstein–Barr virus (EBV), a human transforming gammaherpesvirus, hijacked autophagy during the lytic phase of its cycle, possibly to favor the formation of viral particles. However, the viral proteins that are responsible for an EBV-mediated subversion of the autophagy pathways remain to be characterized. Here we provide the first evidence that the BALF0/1 open reading frame encodes for two conserved proteins of the Bcl-2 family, BALF0 and BALF1, that are expressed during the early phase of the lytic cycle and can modulate autophagy. A putative LC3-interacting region (LIR) has been identified that is required both for BALF1 colocalization with autophagosomes and for its ability to stimulate autophagy.

## 1. Introduction

Autophagy is a homeostatic “self-eating” process involved in the degradation and recycling of cytoplasmic components through a lysosomal-dependent pathway. Autophagy was first identified as a physiological pathway that promotes cell survival [1]. In addition to its role as a metabolic and intracellular biomass and organelle quality and quantity control pathway, autophagy also acts as a microbial clearance mechanism that protects eukaryotic cells against intracellular pathogens. Autophagy also emerged as an alternative pathway to present microbial antigens to the immune system [2]. Accordingly, some pathogens have evolved successful strategies to escape immune control or promote their replication by manipulating autophagy for their own benefit [3,4]. 

Epstein–Barr virus (EBV) is a human enveloped DNA virus from the Herpesviridae family [5]. EBV primary infection occurs usually during childhood with no apparent symptoms, whereas it can be associated with infectious mononucleosis in young adults. EBV establishes a latent, lifelong, persistent infection in more than 95% of the adult population. Although it is usually tightly controlled by the immune system, EBV persistence has been related to a number of malignancies, including some forms of Burkitt’s lymphoma, Hodgkin’s disease, and post-transplant lymphoproliferative diseases as well as epithelial tumors such as undifferentiated nasopharyngeal carcinoma (NPC) and gastric carcinomas [6,7,8,9]. EBV expression patterns alternate latency programs that ensure persistency mainly in B lymphocytes, and lytic phases that allow the production of virions from B lymphocytes and epithelial cells [10]. The induction from latency to the lytic cycle is called reactivation. The differentiation of B cells into plasma following B cell receptor engagement is the most likely physiological stimulus that reactivates EBV from B lymphocytes in vivo [11]. Conversely, many chemical or biological stimuli can be used to reactivate EBV in cultured cell lines, including phorbol esters [12], calcium ionophores [13], transforming growth factor-beta (TGF-β) [14], and sodium butyrate [15], hypoxia [16], oxidative stress [17], or following activation of B cell receptor with antibodies directed against surface immunoglobulins (anti-sIg) [18].

Recent studies showed that EBV could modulate autophagy during both latency and reactivation. During latency, latent membrane protein 1 (LMP1) induces autophagy to control its own degradation [19], latent membrane protein 2 (LMP2A) induces autophagy to promote abnormal acinus formation [20], and EBV nuclear antigen 3C (EBNA3C) activates autophagosome formation through transcriptional induction of several autophagy regulators including ATG3, ATG5, and ATG7 [21]. During the EBV lytic cycle, autophagy has been proposed to be modulated in a complex bimodal way that combines stimulation of the early phase (i.e., autophagosome formation) with inhibition of the latest phase (i.e., degradation of autophagosome content following the fusion between autophagosomes and lysosomes). Accordingly, De Leo and colleagues showed that autophagy was transiently induced following EBV reactivation and then inhibited during the latest step of the lytic cycle [22]. Blocking autophagy at the final step may possibly favor the acquisition of viral envelopes and components of the autophagic machinery by the neosynthesized virions [23,24]. Except for Rta, an immediate-early protein that stimulates the expression of autophagy-related genes through an ERK-dependent pathway [25], the viral proteins that modulate autophagy during the lytic cycle are still poorly characterized. 

In the present work, we wondered whether EBV proteins whose viral or cellular orthologs modulate autophagy might also modulate this process. Cellular Bcl-2 was initially discovered in acute lymphoblastic leukemia [26] and later shown to protect cells from apoptosis [27]. Bcl-2 and two *Herpesviridae*-encoded Bcl-2 orthologs, M11 (murine γ-herpesvirus 68, MHV68), and Ks-Bcl-2 (Kaposi’s sarcoma herpesvirus, KSHV), inhibit autophagy through their interaction with Beclin 1, a cellular protein that is required for the initiation of autophagosome formation [28,29]. Interestingly EBV encodes for two viral Bcl-2 homolog (vBcl-2) proteins, BHRF1 and BALF0/1 [30,31]. Both BHRF1 and BALF0/1 prevent apoptosis during early infection of primary B cells but may be dispensable once a latent infection is established [32]. While BHRF1 anti-apoptotic activity has been extensively studied [33], the expression and function of BALF0/1 are still equivocal. Indeed, two in-frame methionine codons are present near the beginning of the BALF0/1 open reading frame (ORF), suggesting that two proteins with different N-termini may be encoded [34]. BALF1 protein would be encoded by the shorter ORF, while the protein encoded from the first nonconserved methionine is referred to as BALF0. BALF0/1 is transcribed in both lytic stage and latency in EBV-positive Burkitt lymphoma’s cell lines and NPC biopsies [35]. Ectopic expression of BALF0/1 promotes tumor formation and metastasis in nude mice [36]. While it was initially suggested that BALF0/1 could inhibit apoptosis by interacting with Bax and Bak [31], Bellows and co-workers showed that BALF1 failed to protect against Sindbis virus- or Bax-induced apoptosis and antagonized the anti-apoptotic activity of BHRF1 [34]. BALF0 also antagonized the anti-apoptotic activity of BHRF1 but did not associate with either BHRF1 or BALF1 [34]. Most importantly, the existence of BALF0 and/or BALF1 in cells naturally infected by EBV has never been confirmed due to the absence of specific antibodies. Previous work has shown that NPC patients may produce antibodies recognizing a 31 kDa protein in BALF0/1-transfected NIH3T3 cells [35], which was compatible with BALF0 expected size. Nonetheless, the existence of BALF1 could not be confirmed in the same study. More recently, we designed an ELISA to detect anti-BALF0/1 antibodies in human plasma samples. This assay led to the detection of low-titer immunoglobulin Gs (IgGs) to BALF0/1 during primary (10.0%) and past (13.3%) EBV infection, whereas high-titer IgGs could be detected in 33.3% of patients with NPC. Thus, this latest work provided important indirect evidence for the expression of BALF0 and/or BALF1 in vivo [37]. 

In the present study, we were able to confirm that both BALF0 and BALF1 are expressed in naturally infected B cells and provide evidence that BALF0 and BALF1 could modulate autophagy.

## 2. Materials and Methods

### 2.1. Sequence Alignment

All the sequences used in this study are listed in Supplementary Table S1. A search for proteins homologous to BALF1 and BALF0 was performed on the National Center for Biotechnology Information (NCBI) database (www.ncbi.nlm.nih.gov). Alignments of protein sequences were performed using the ClustalW method using MacVector (version 17.0.2) with default settings. Phylogenetic trees were generated using the unweighted pair group method with arithmetic mean (UPGMA) by MacVector (version 17.0.2).

### 2.2. Cell Culture, Treatment, and Transient Transfection

HeLa cells and the EBV-positive Burkitt’s lymphoma cell line Akata (purchased from the American Type Culture Collection (ATCC)) were cultured at 37 °C under 5% CO_2_ in RPMI-1640 (Gibco, Illkirch, France) supplemented with 10% fetal calf serum (FCS; Thermo Scientific, Illkirch, France). HeLa cells with stable expression of GFP-LC3 and mRFP-GFP-LC3 were kindly provided by Aviva Tolkovsky [38] and David Rubinsztein [39], respectively. These cells were grown in RPMI-1640 supplemented with 10% FCS and G418 (500 µg/mL; Invivogen, Toulouse, France). In order to induce the EBV lytic cycle, Akata cells were treated with 7.5 µg/mL of polyclonal rabbit anti-human IgG (Dako, Santa Clara, USA). Autophagic flux was monitored by the addition of chloroquine (50 μM) 4 h prior to cell lysis. Starvation-induced autophagy was carried out by replacing the complete medium with Earle’s Balanced Salt Solution (EBSS; Gibco) for 4 h before performing immunofluorescence staining. DNA plasmid transfections were performed by using Fugene HD transfection reagent (Promega, Madison, USA) according to the manufacturer’s instructions. 

### 2.3. RNA Extraction and qRT-PCR

To determine the relative mRNA expression of BALF0/1, Akata cells were harvested at the indicated time post-induction and total RNA was extracted by using TRIzol reagent (Invitrogen). Five micrograms of purified total RNA were treated with DNase from a TURBO DNA-free kit (Life Technologies). Then 2 µg of DNase-treated RNA was reverse-transcribed into cDNA by a High-Capacity cDNA Reverse Transcription Kit (Thermo Scientific). Transcriptional expression was measured by quantitative real-time PCR (qRT-PCR) with a KAPA SYBR Fast Universal Readymix Kit (Kapa Biosystems, Wilmington, USA), and specific primers are listed in Supplementary Table S2. Cellular cyclophilin was used as a control to normalize viral mRNA expression. Experiments were performed on the CFX96 Touch Real-Time PCR Detection System (Bio-Rad) and analyzed with the 2^−ΔΔC_T_^ method [40]. 

### 2.4. Plasmid Construction and Mutagenesis

The DNA sequence corresponding to the open reading frame of BALF0/1 was amplified by PCR using viral genomic DNA (EBV B95.8 strain) as a template. Primers were designed according to the manufacturer’s instructions for the Cold Fusion Cloning Kit (SBI, Palo Alto, USA). The sequence encoding the hemagglutinin (HA) tag was added to the reverse primer to generate a C-terminal HA-tag fused with BALF0/1 from the expression vector pcDNA3.1 (Invitrogen). The amplified PCR product was inserted into the linearized pcDNA3.1 (EcoRI, NEB, Évry, France) by ligation using the Cold Fusion Cloning Kit. Mutant constructions were generated by site-directed mutagenesis from the template pcDNA3.1-BALF0/1-HA. All PCRs for plasmid construction were performed under standard conditions by using *PfuUltra* DNA polymerase (Agilent Technologies, Santa Clara, USA) and plasmids were verified by sequencing. The sequences of primers for plasmid construction and mutagenesis are listed in Supplementary Table S2. 

### 2.5. Immunoblotting

Transfected cells were collected at 48 h post-transfection and reactivated Akata cells were harvested at the indicated time. Cell pellets were lysed in lysis buffer (50 mM Tris·HCl pH 6.8, 2% SDS, 2% β-mercaptoethanol), subjected to SDS-PAGE, and transferred onto a polyvinylidene fluoride (PVDF) membrane (Amersham). The membranes were blocked with 5% bovine serum albumin (BSA) or skim milk powder and incubated at 4 °C overnight with the indicated antibodies. Anti-ZEBRA (sc-53904; 1/5000), anti-β-actin (sc-47778; 1/5000), and anti-HA (sc-805; 1/1000) antibodies were purchased from Santa Cruz; anti-LC3B (L7543) (1/4000) and anti-Sequestosome1 (SQSTM1/p62) (5114T; 1/4000) were obtained from Sigma and Cell Signaling Technology, respectively. Anti-sera against BALF0/1 were prepared by immunizing a rabbit with the recombinant protein of BALF0/1, produced as previously described [37], and used for immunoblotting analysis at a dilution of 1/500. Horseradish peroxidase-conjugated goat antibodies directed against mouse (Cell Signaling Technology, Leiden, Netherlands) or rabbit (Amersham, Saint-Quentin Fallavier, France) immunoglobulins were used as secondary antibodies (1/10,000). Immunodetection was performed using the ECL detection system according to the manufacturer’s instructions (Amersham). 

### 2.6. Immunofluorescence Analysis

Cells were grown on 8-well Lab-Tek chamber slides (Thermo Scientific) and fixed 24 h after transfection with paraformaldehyde (4%) in phosphate-buffered saline (PBS) for 10 min at room temperature (RT). Fixed cells were washed with PBS twice and permeabilized with 0.2% Triton X-100 for 5 min at RT, blocked with 5% FCS, and incubated with anti-HA rabbit antibody (1/100) or anti-BALF0/1 rabbit sera (1/200) for 1 h at 37 °C. Then, the cells were washed with PBS and incubated with the secondary antibody at a dilution of 1/1000 (Alexa Flour 555 goat anti-rabbit IgG or Alexa Flour 647 goat anti-rabbit IgG, Thermo Scientific). Next, the cells were washed with PBS and the nuclei were counterstained with Hoechst 33342 (Thermo Scientific). Coverslips were mounted in Glycergel mounting medium (Dako) and observed by using a Zeiss AxioObserver Z1 or Leica SP8 confocal laser microscope. Images were resized, organized, and labeled using ImageJ software. Three-dimensional reconstruction was established by IMARIS (Bitplane, Belfast, UK) software. 

### 2.7. Statistics

Data from 3 independent experiments are presented as mean ± standard error of the mean (SEM), which were analyzed with Prism software (GraphPad, San Diego, USA) by using Student’s *t*-test or one-way analysis of variance (ANOVA) test comparisons. P values less than 0.05 were considered statistically significant. 

## 3. Results

### 3.1. BALF0 and BALF1 Are Both Expressed during EBV Reactivation and Cross-Regulate Each Other

**Figure 1:**
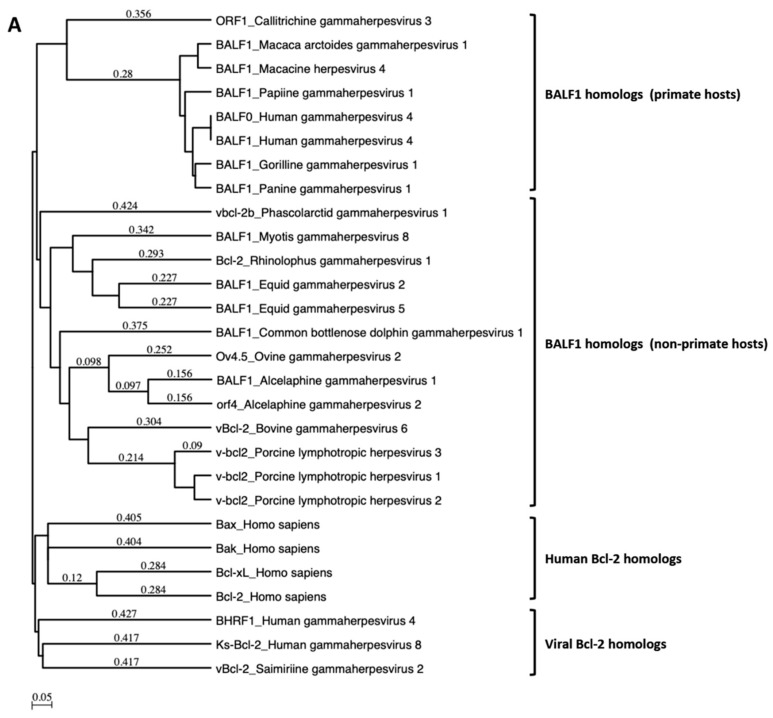

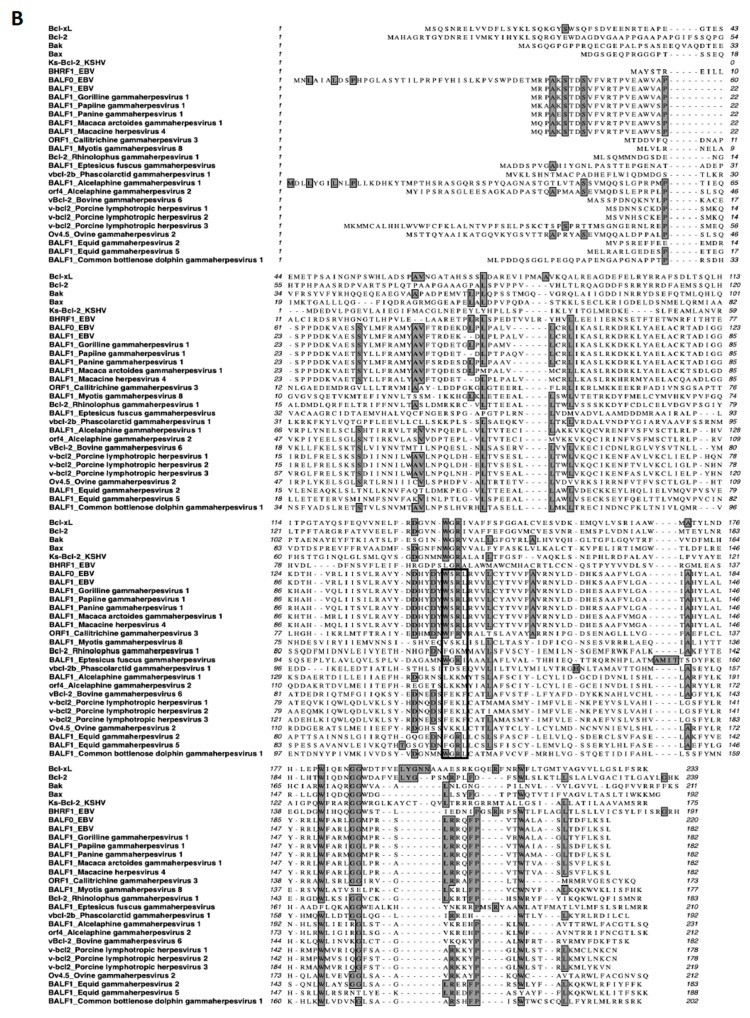
BALF1 of primate and non-primate herpesviruses. (**A**) Phylogenetic tree generated using an unweighted pair group method with arithmetic mean (UPGMA) from amino acid sequences of indicated human and viral Bcl-2 family members as well as BALF1 from primate and non-primate herpesviruses. (**B**) ClustalW alignment of amino acid sequences analyzed in (**A**). Identical amino acids are marked in dark shading. The putative LC3-interacting region (LIR) motif of BALF1 is marked by a box. GenBank accession numbers of sequences used in this study are listed in Supplementary Table S1. The analysis was performed by MacVector software.

**Figure 2:**
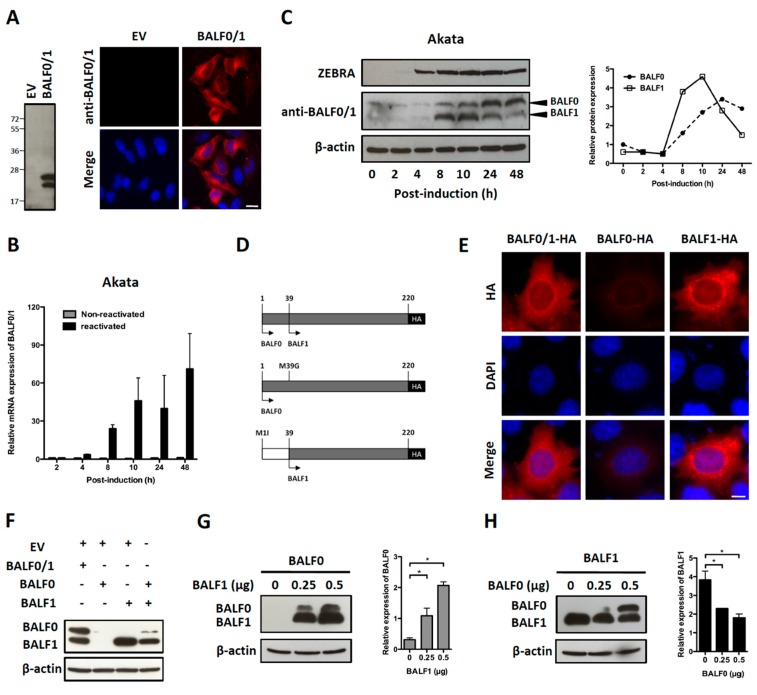
Characterization of BALF0 and BALF1 expression. (**A**) Characterization of rabbit anti-sera against BALF0/1. HeLa cells were transfected with a plasmid encoding for BALF0/1 (pcDNA3.1-BALF0/1-HA) or corresponding negative control (empty vector, EV). BALF0/1 expression was analyzed at 48 h post-transfection (p.t.) by immunoblot (left panel) and immunofluorescence (right panel) using rabbit anti-sera directed against BALF0/1. Scale bar = 20 µm. Two polypeptides whose relative mobility following SDS-PAGE corresponded to the predicted size of BALF0 (26 kDa) and BALF1 (22 kDa) were detected by immunoblot. (**B**) Time-course accumulation of BALF0/1 mRNA in reactivated Akata cells. Akata cells were reactivated by cross-linking of surface immunoglobulin for 2 to 48 h. At the indicated time post-reactivation, mRNA encoding for BALF0/1 was quantified by qRT-PCR. (**C**) BALF0 and BALF1 protein expression in reactivated Akata cells. Total protein was extracted from Akata cells as described in (**B**) and analyzed by immunoblot (left panel) using rabbit anti-sera against BALF0/1. Immediate-early protein ZEBRA was used as a marker for viral reactivation. Relative expression of BALF0 and BALF1 was analyzed using ImageJ (right panel) and compared to β-actin loading control at each time point. (**D**) Schematic diagram of expression vectors encoding for BALF0/1 (pcDNA3.1-BALF0/1-HA), BALF0 (pcDNA3.1-BALF0-HA), and BALF1 (pcDNA3.1-BALF1-HA). BALF0 and BALF1 alone were obtained by replacing the methionine at amino acid 39 and 1 with glycine and isoleucine, respectively. (**E**) Immunofluorescence staining of HA-tagged BALF0/1, BALF0, and BALF1. HeLa cells were analyzed 24 h p.t. by immunofluorescence using an anti-HA antibody. Scale bar = 10 µm. (**F**) Immunoblot analysis of BALF0/1, BALF0, and BALF1. HeLa cells were co-transfected with 0.25 μg of each indicated plasmid for a total plasmid amount of 0.5 μg. Protein expression was analyzed 48 h p.t. by immunoblot using rabbit anti-sera against BALF0/1. (**G**,**H**) Cross-regulation of BALF0 and BALF1 expression. HeLa cells were transfected with a constant amount (0.25 μg) of BALF0 (**G**) or BALF1 (**H**) encoding plasmid in the presence of increasing amount of BALF1 (**G**) or BALF0 (**H**) encoding plasmid. EV plasmid was added to keep the total amount of transfected plasmids constant. Immunoblot analysis was carried out as described in (**F**). Relative expression of BALF0 and BALF1 was evaluated by densitometric analysis by ImageJ and compared to that of β-actin loading control. Values are mean ± SEM of three independent experiments. One representative set of immunoblotting results is shown. * *P* < 0.05.

A search for proteins homologous to BALF1 and BALF0 was performed on the NCBI database. This led to the identification of six homologous proteins in gammaherpesviruses from primates that were closely related to EBV BALF1 and BALF0, as well as 13 proteins of gammaherpesviruses from non-primate hosts (Figure 1A). Interestingly, BALF0 and BALF1 from EBV were more closely related to BALF1 from other gammaherpesviruses than to EBV BHRF1, which belongs to a homology group containing vBcl-2s from herpesvirus saimiri and Kaposi’s sarcoma herpesvirus (KSHV). BALF0/1 ORF encodes for a putative 220-amino-acid protein, BALF0. Due to the presence of a conserved internal putative start codon at position 39, this ORF is also predicted to encode for a 182-amino-acid polypeptide referred to as BALF1 (Figure 1B). Sequence alignment revealed that amino acids 1 to 38 from BALF0 were unique to Epstein–Barr virus among BALF1 homologs from primate gammaherpesviruses (Figure 1B). Due to the lack of specific antibodies, the existence of BALF0 and BALF1 proteins has not been assessed in naturally infected cells so far. To overcome this limit, we generated a specific polyclonal antiserum directed against a recombinant form of BALF0/1. For this purpose, a truncated form of BALF0 encoding for amino acids 1 to 140 was expressed and purified from *Escherichia coli* [37] and used as an antigen to obtain rabbit anti-BALF0/1 antibodies. The resulting antiserum specifically detected polypeptides whose size was compatible with BALF0 and BALF1 following immunoblotting analysis of HeLa cells transfected with pcDNA3.1-BALF0/1-HA, an expression vector expressing BALF0/1 mRNA (Figure 2A left panel). BALF0 and/or BALF1 were also detected by immunofluorescence in the cytoplasm of transfected cells as previously reported (Figure 2A, right panel) [34].

Akata is a Burkitt’s lymphoma-derived cell line in which EBV established a type I latent infection. EBV can be reactivated from Akata cells following B cell receptor cross-linking with anti-surface immunoglobulins [41]. In a first attempt to investigate the BALF0/1 expression pattern, Akata cells were reactivated and BALF0/1 mRNA was analyzed by qRT-PCR. In agreement with previous work [35], BALF0/1 mRNA was barely detectable in non-reactivated cells but progressively accumulated from 8 to 48 h following reactivation (Figure 2B). Immunoblot analysis demonstrated that both BALF0 and BALF1 could be detected at very low levels in non-reactivated cells. BALF0 and BALF1 were mainly expressed during early lytic infection and accumulated shortly after the expression of ZEBRA, an immediate-early protein that is essential for the virus to enter the lytic cycle (Figure 2C) [42]. Surprisingly, BALF1 accumulation preceded BALF0’s, whereas BALF0 accumulation was conversely associated with a marked decrease in the level of BALF1 (Figure 2C). This suggested that BALF0 and BALF1 synthesis and/or degradation could be cross-regulated. To test this hypothesis, expression vectors encoding for HA-tagged BALF0 and BALF1 (pcDNA3.1-BALF0/1-HA), BALF0 alone (pcDNA3.1-BALF0-HA), and BALF1 alone (pcDNA3.1-BALF1-HA) were constructed by mutating the first (BALF1) or second (BALF0) start codon (Figure 2D). Comparable amounts of BALF0 and BALF1 were expressed in HeLa cells transfected with pcDNA3.1-BALF0/1-HA. While BALF1 was expressed at a high level from pcDNA3.1-BALF1-HA, BALF0 was barely detectable when expressed alone from pcDNA3.1-BALF0-HA either by immunofluorescence (Figure 2E, lane 2) or immunoblot (Figure 2F, lane 2). This led us to hypothesize that BALF1 expression could promote BALF0 accumulation. This was further confirmed since BALF0 accumulation was at least partly restored when pcDNA3.1-BALF0-HA was co-transfected with pcDNA3.1-BALF1-HA (Figure 2F). To confirm this result, we analyzed BALF0 expression from pcDNA3.1-BALF0-HA in the presence of increasing amounts of pcDNA3.1-BALF1-HA. As expected, BALF0 accumulation positively correlated with the amounts of pcDNA3.1-BALF1-HA (Figure 2G). Conversely, transfecting increasing amounts of pcDNA3.1-BALF0-HA in the presence of a constant amount of pcDNA3.1-BALF1-HA led to a significant decrease in BALF1 accumulation (Figure 2H). Since the total amount of transfected plasmids was constant in each experiment, it was concluded that BALF1 promoted BALF0 accumulation, which in turn inhibited BALF1 expression, therefore providing a possible explanation for the unbalanced kinetics of both proteins during EBV reactivation.

### 3.2. BALF1 and BALF0/1 Modulate Autophagy

Previous work showed that cellular Bcl-2 could inhibit autophagy. Indeed, Bcl-2 interacts with Beclin 1, which prevents Beclin 1 from assembling the pre-autophagosomal structure [43]. Since BALF0 and BALF1 share noticeable homology with cellular Bcl-2, we wondered whether they might modulate autophagy as well [28]. Considering that BALF1 and BALF0 were sequentially expressed during EBV reactivation, autophagy was analyzed in HeLa cells either expressing BALF1 alone or co-expressing BALF0 and BALF1. Due to the low expression of BALF0, its impact on autophagy could not be measured by immunoblot analysis of autophagy markers.

**Figure 3:**
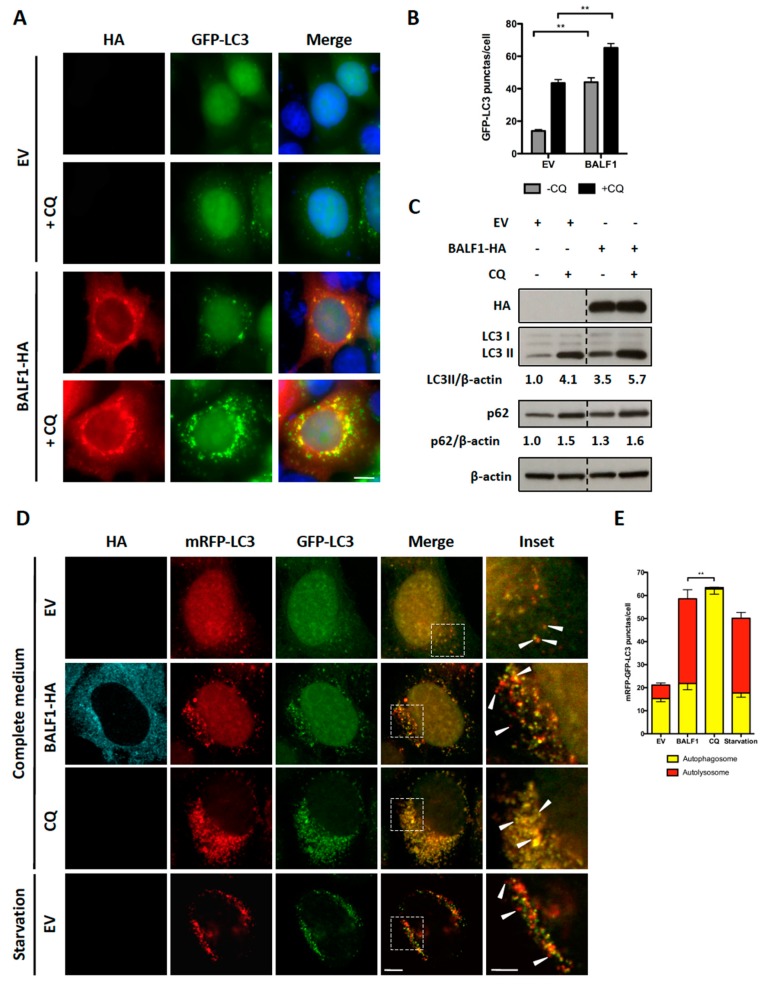
BALF1 stimulates the formation of autophagosomes and autophagic flux in HeLa cells. (**A**) Representative images of GFP-LC3 HeLa cells transfected with EV- or BALF1-HA-encoding plasmids. Cells were cultured in complete medium with or without chloroquine (CQ) 24 h p.t. Ectopic expression of BALF1 was detected by anti-HA antibody and observed by fluorescence microscopy. Scale bar = 20 µm. (**B**) Autophagosome formation was evaluated by counting the number of GFP-LC3 puncta per cell; 50 cells were analyzed per assay and results are means ± SEM of three independent experiments. ** *P* < 0.01. (**C**) Immunoblot analysis of cellular LC3 and p62 proteins in HeLa cells transfected with EV or BALF1-HA plasmids. Cells were cultured in complete medium with or without CQ 48 h p.t. β-actin was used as a loading control. LC3-II and p62 to β-actin ratios were evaluated by densitometric analysis using ImageJ. (**D**) HeLa cells stably expressing mRFP-GFP-LC3 were transfected with EV- and BALF1-HA-encoding plasmids, starved or treated with CQ. Cells were analyzed by immunofluorescence 24 h p.t. and analyzed by confocal microscopy. Scale bar = 20 µm and 5 µm for insets. (**E**) Quantification of yellow puncta (mRFP-GFP-LC3 positive) and red puncta (mRFP-LC3 positive) per cell. Values represent means ± SEM of three independent experiments; 50 cells were analyzed in each assay. ** *P* < 0.01.

Microtubule-associated protein light chain 3 (LC3) is a widely used marker for autophagosomes. During autophagy, cytosolic LC3-I is conjugated to phosphatidylethanolamine to form LC3-II, which is subsequently incorporated into the autophagosomal membrane. Therefore, LC3-II level positively correlates with the number of autophagosomes. To investigate the impact of BALF1 on autophagosome formation, BALF1-HA expression vector was transfected into HeLa cells that stably expressed a GFP-labeled form of LC3B (GFP-LC3). In these cells, autophagosomes and autolysosomes appear as small cytoplasmic vesicles. Chloroquine (CQ) neutralizes the lysosomal pH and causes the accumulation of GFP-LC3-positive vesicles by inhibiting endogenous protein degradation [44]. As illustrated in Figure 3A,B, BALF1 induced a significant increase in the number of GFP-LC3-containing vesicles, suggesting that BALF1 either stimulates autophagosome formation or alternatively inhibits the fusion of autophagosomes with lysosomes. Importantly, we noticed that BALF1 concentrated in GFP-LC3 formed positive puncta. To explore autophagic flux, which reflects autophagic degradation activity, we analyzed the accumulation of LC3-II by immunoblot in cells that were treated with or without CQ. As shown in Figure 3C, BALF1 expression induced a significant increase in LC3-II that was markedly amplified in the presence of CQ, indicating that BALF1 indeed stimulates autophagic flux. Next, we used an expression vector encoding for a tandem fluorescent-tagged LC3 (mRFP-EGFP-LC3B) probe. This probe is a convenient tool for monitoring autophagic flux based on the fact that EGFP and mRFP fluorescent proteins have different stability in response to low pH. Indeed, the fusion between autophagosomes and lysosomes is associated with a decrease in pH that affects EGFP but not mRFP fluorescence [45]. As a result, autophagosomes are dually labeled with EGFP and mRFP, whereas acidic autolysosomes are only labeled with mRFP. As illustrated in Figure 3D,E, CQ inhibited pH decrease, which induced the accumulation of vesicles labeled with both mRFP and EGFP (autophagosomes). On the contrary, BALF1-HA induced the concomitant accumulation of both dually labeled (autophagosomes) and red-only (autolysosomes) vesicles, confirming that it significantly increased autophagy flux up to the formation of autolysosomes. 

**Figure 4:**
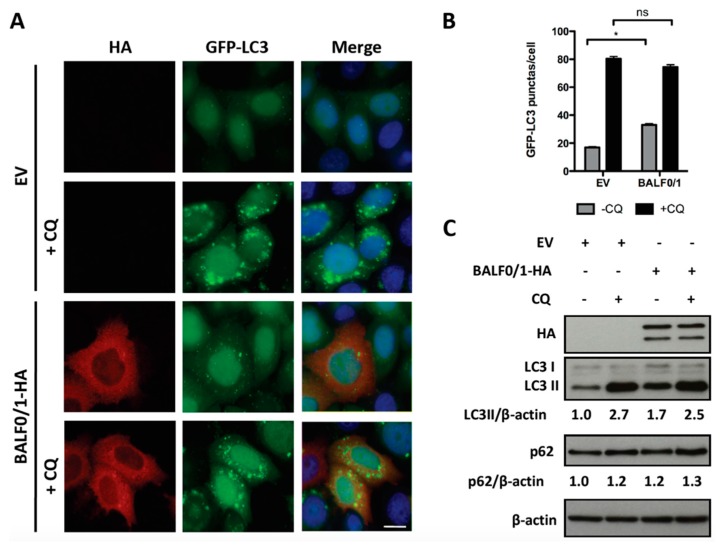
BALF0 limits the activity of BALF1 in autophagy. (**A**) HeLa cells stably expressing GFP-LC3 were transfected with EV or BALF0/1 plasmid. The analysis was performed 24 h after transfection. When indicated, cells were treated for 4 h by CQ. BALF0/1-transfected cells were visualized by anti-HA antibody (red), and nuclei were stained with Hoechst 33342 (blue). (**B**) The number of GFP-LC3 puncta was quantified. Results are means ± SEM of three independent experiments; 50 cells were analyzed per assay. Ns, not significant; * *P* < 0.05. (**C**) Immunoblot analysis of cellular LC3 and p62 proteins in HeLa cells transfected with EV- or BALF0/1-HA-encoding plasmids. Cells were cultured in complete medium with or without CQ (4 h) 48 h p.t. β-actin was used as loading control. LC3-II and p62 to β-actin ratios were evaluated by densitometric analysis by ImageJ.

Since it was previously demonstrated that BALF0 limited BALF1 accumulation, we wondered whether the stimulation of autophagy by BALF1 was similar in the presence of BALF0. For this purpose, autophagy was investigated in HeLa cells expressing both proteins from the same vector (pcDNA3.1-BALF0/1-HA). As before, BALF0/1 expression was associated with an increase in the number of GFP-LC3-positive puncta (Figure 4A,B) as well as LC3-II accumulation (Figure 4C), albeit to a lesser extent compared to BALF1 alone. This effect, however, could not be observed anymore in the presence of CQ (in comparison to empty vector and pcDNA3.1-BALF0/1-HA in the presence of CQ), indicating that the autophagic flux induced by BALF1 was reduced in the presence of BALF0. Due to the very low expression level of BALF0, when expressed alone, its impact on autophagy could not be evaluated by LC3 or p62 Western blot. However, we could confirm that BALF0 induced the formation of autophagosomes in HeLa cells stably expressing GFP-LC3 (Supplementary Figure S1).

Altogether, these results led to the conclusion that BALF1 stimulates autophagic flux, which, in turn, is limited in the presence of BALF0. In both instances, the expression of BALF1 alone or in association with BALF0 was associated with the accumulation of Sequestosome1 (SQSTM1/p62). SQSTM1/p62 is a multifunctional protein that is notably involved in the clearance of aggregates by autophagy [46]. p62 binds directly to LC3 and GABARAP family proteins and is degraded following fusion between phagosomes and lysosomes. Therefore, p62 accumulates when autophagy is inhibited and is degraded when autophagy is stimulated up to the protein degradation step. For this reason, p62 has long been used as a marker to study autophagy-mediated proteolysis [47]. As shown in Figure 3C and Figure 4C, p62 slightly accumulated in BALF1- and BALF0/1-expressing cells in the absence of chloroquine (compare p62/β-actin ratio between lanes 1 and 3), which suggested that BALF1-mediated increase in autophagosome formation was not associated with an increase in autophagy-mediated proteolysis. This was also confirmed by the accumulation of LC3, another target of autophagy-mediated degradation.

### 3.3. LC3-interacting region (LIR)-Like Motif Is Required for BALF1 Stimulation of Autophagic Flux

**Figure 5:**
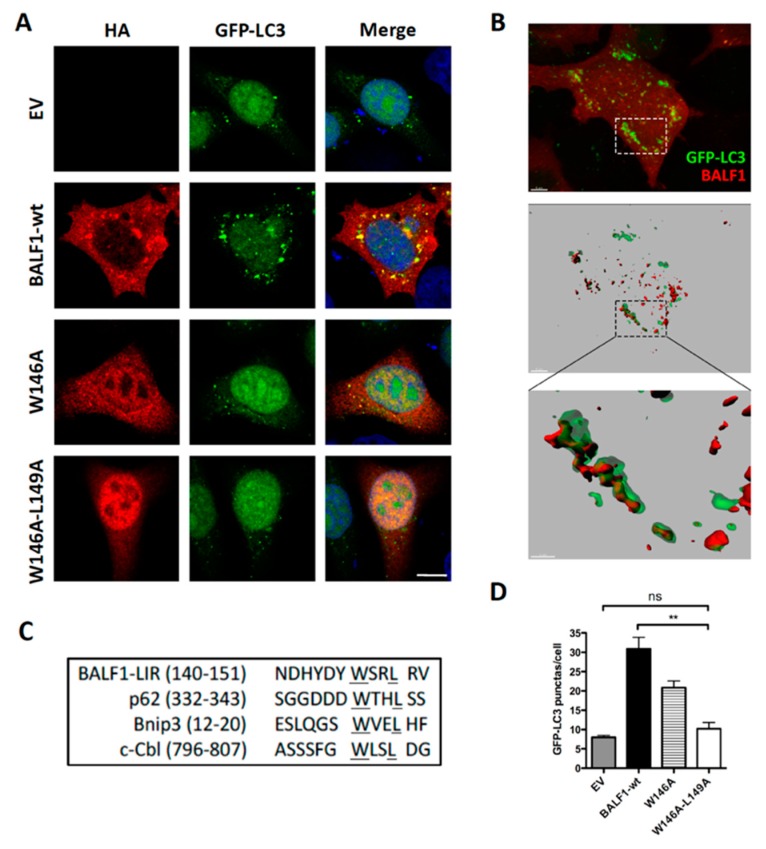
BALF1 modulates autophagy through an LIR-like motif. (**A**) Representative images of GFP-LC3 HeLa cells transfected with EV and plasmids encoding for BALF1-HA or the indicated BALF1 mutants. At 24 h p.t., cells were analyzed by immunofluorescence and observed by confocal microscopy. (**B**) GFP-LC3 HeLa cells transiently transfected with BALF1-HA. 3D volume rendering (top) and relative IMARIS isosurface 3D rendering (middle) are shown. Scale bar = 5 µm. Magnification of the boxed area is shown in the inset at the bottom (scale bar = 2 µm). (**C**) Typical LIR sequences were aligned alongside BALF1. Underlined regions indicate highly conserved residues in the putative LIR motif of BALF1. (**D**) GFP-LC3 puncta were quantified with ImageJ in cells transfected with plasmids encoding for wild-type (wt) or LIR-like mutants of BALF1. Mean ± SEM; *n* = 50 cells from three independent experiments; ns, not significant; ** *P* < 0.01.

BALF0/1 proteins were previously described as diffuse cytoplasmic proteins [34]. Since B cells have reduced cytoplasmic volume, BALF0/1 subcellular localization was investigated herein by expressing HA-tagged proteins in epithelial HeLa cells. BALF0/1-HA (Figure 2E) as well as BALF0-HA and BALF1-HA mainly accumulated in the cytoplasm, although low levels of BALF0-HA and BALF1-HA could also be detected in the nucleus. Confocal microscopy analysis revealed that BALF1 (Figure 5A) and BALF0 (Supplementary Figure S2) could also form discrete cytoplasmic puncta that colocalized with GFP-LC3-positive vesicles and with endogenous LC3 containing vesicles [48]. Z-stacked confocal images were collected, and three-dimensional reconstruction was performed to more precisely investigate BALF1 and LC3 subcellular colocalization. As illustrated in Figure 5B, BALF1- and GFP-LC3-positive vesicles were in close contact or overlapped each other. This led us to hypothesize that BALF1 might be addressed to GFP-LC3-containing vesicles, possibly through an LC3-interacting region (LIR) motif. Protein complexes are recruited to autophagosomes through an LIR motif, to act as either autophagy adaptors or receptors. Autophagy receptors interact with mammalian ATG8 family proteins on the inner autophagosomal membrane, which allows the cargo to be targeted to the lysosomal pathway for degradation. Conversely, autophagy adaptors interact with ATG8 family proteins on the convex autophagosomal membrane surface, where they regulate autophagosome formation, fusion with the lysosome, or autophagosome transport. Importantly, adaptors are not degraded by autophagy [49,50]. A subset of verified LIR motifs led to the identification of a core consensus sequence [W/F/Y]xx[L/I/V], which consists of four key amino acids including an aromatic residue (W/F/Y) at the first position and a hydrophobic residue (L/I/V) at the fourth position, while x may be any other residue [51,52]. Sequence analysis of BALF1 revealed the presence of a putative LIR motif between amino acids 146 to 149 (146-WSRL-149) that closely resembles known LIR motifs (Figure 5C). This putative LIR motif was highly conserved among BALF1 orthologs from primate gammaherpesviruses (Figure 1B, boxed amino acids), with the noticeable exception of BALF1 from Callitrichine gammaherpesvirus 3, in which the second and fourth amino acids were different. Nonetheless, the resulting sequence (WFRV) still matched the consensus LIR motif. To evaluate its effective contribution to BALF1 targeting to LC3-containing vesicles, discrete mutations were generated in which one (W146A) or two (W146A and L149A) essential amino acids of the putative LIR motif were modified. As shown in Figure 5A, these mutations had a dramatic effect on the subcellular localization of BALF1, inducing either a partial (W146A) or total (W146A and L149A) relocalization of the modified proteins into the nucleus. To evaluate the impact of these mutations on BALF1’s ability to stimulate autophagy, we measured the average number of autophagosomes (GFP-LC3 puncta) in HeLa cells expressing BALF1 single (W146A) or double (W146A/L149A) mutant. As shown in Figure 5A,D, these mutations dramatically reduced BALF1’s ability to promote autophagosome formation. These experiments led to the conclusion that this region is required both for efficiently targeting BALF1 to GFP-LC3 vesicles and for BALF1’s ability to promote autophagy.

## 4. Discussion

The majority of herpesviruses examined so far have evolved various strategies to manipulate autophagy, either to improve their persistence or to optimize their replication [53]. Previous studies indicated that EBV reactivation and autophagy were intimately involved, possibly in a bimodal way. On the one hand, early steps of autophagy are induced during EBV reactivation, and molecules that can inhibit autophagy such as chloroquine, ammonium chloride (Quignon et al., in prep), or 3-methyladenine [25] repress the accumulation of lytic proteins and reduce the production of viral particles. On the other hand, the final steps of autophagy, i.e., the fusion between autophagosomes and lysosomes and the subsequent degradation of cargo within autolysosomes, are inhibited [23]. Taken together, these processes may eventually lead to the accumulation of autophagic material, such as autophagic machinery components and vesicle membranes, that could be used to produce viral particles. Concomitantly, this would limit the degradation of viral components as well as viral particles by autophagy. In agreement with this model, Nowag and co-authors provided the first evidence that LC3-associated membranes might be readdressed to virion envelopes [24]. In a first attempt to identify EBV proteins that could modulate autophagy during reactivation, we tested whether lytic proteins whose cellular or viral orthologs were known to affect autophagy could also affect autophagy. During lytic replication, herpes simplex type 1 (HSV-1) [54], MHV68 [55], KSHV, [28], and human cytomegalovirus (HCMV) [56,57] encode for proteins that can inhibit early steps of autophagy by binding to and inactivating Beclin-1, a cellular protein that is required for phagophore formation [53]. Since Beclin-1 was demonstrated to be targeted and inhibited by cellular Bcl-2 [28], we tested whether Bcl-2 orthologs from EBV, namely BHRF1, BALF0, and BALF1, could modulate autophagy as well. Database analysis showed that orthologs of BALF0 and BALF1 can be identified in most known gammaherpesviruses. In addition, BALF0 and BALF1 proved to be more related to each other than to EBV BHRF1, KSHV vBcl-2 (Ks-Bcl-2), and Saimiriine gammaherpesvirus 2 (Figure 1). Although BALF1 orthologs from primate hosts were closely related, we could not identify BALF0 orthologs even in the closest relatives of EBV BALF1 proteins, as previously reported [34]. The production of specific antibodies to BALF0/1 led to the detection of both BALF0 and BALF1 during EBV reactivation in Akata cells. BALF0 and BALF1 proteins were also detected in B95.8 cells (latency III), albeit at low levels. Surprisingly, BALF0 and BALF1 only mildly accumulated following reactivation in these cells [58]. Using plasmids encoding both proteins from the same ORF or separately, we demonstrated that BALF1 (Figure 3) and BALF0 (Supplementary Figure S1) increased the number of autophagosomes when expressed individually. This correlated with the accumulation of the membrane-bound form of LC3, which is the core autophagic machinery required for elongation of the phagophore. Importantly, the number of LC3-positive vesicles and the accumulation of LC3-II significantly increased in the presence of CQ in BALF1-expressing cells, indicating that BALF1 has a global positive impact on autophagic flux. This result was reminiscent of previous work demonstrating that the adenovirus protein E1B19K could activate autophagy. Indeed, E1B19K, a Bcl-2 ortholog, could replace Bcl-2 in the Beclin-1 complexes, thereby activating PI3KC3 and promoting the early phases of autophagy [59]. Overexpression of both BALF0 and BALF1 resulted in the accumulation of LC3-positive vesicles, but LC3-II did not accumulate in the presence of CQ, suggesting that BALF0 tempered BALF1’s pro-autophagic activity. This agreed with the fact that BALF1 accumulation decreased in the presence of BALF0. Additional experiments showed that both proteins were stabilized in the presence of the proteasome inhibitor MG132 [60]. Thus, it is possible that BALF0 and BALF1 proteins act directly or indirectly on the proteasomal degradation pathway, a hypothesis that is currently under investigation by our group. These results are also in agreement with the unbalanced accumulation of BALF0 and BALF1 that was observed during EBV reactivation and with previous observations by De Leo and colleagues, who observed transient accumulation of LC3-II in Akata cells 8 hours after reactivation [22], a time point where BALF1 accumulated in these cells. The differential impact of BALF0 and BALF1 on autophagy confirms that they may exhibit slightly different biological activities. Accordingly, Bellows and collaborators have already noticed that while both BALF0 and BALF1 could antagonize BHRF1 anti-apoptotic activities, only BALF1 could interact with BHRF1 in vitro [34]. 

Stimulation of autophagy by BALF1 was shown to require a conserved motif (amino acid 146-WSRL-149) that is reminiscent of an LIR motif that has been identified in known partners of LC3, such as Sequestosome1 (SQSTM1/p62) (WTHL). SQSTM1/p62, a multidomain protein, has been identified as the first selective receptor for autophagic degradation of ubiquitylated protein aggregates. SQSTM1/p62 is also a selective autophagy substrate, whose interaction with phagophore membranes is mediated through an LIR domain [61,62,63]. Herein, it is demonstrated that BALF1 expression (as well as co-expression of BALF1 with BALF0) induced accumulation of p62 (Figure 3C and Figure 4C). This suggests that p62 escapes autophagic degradation in the presence of BALF1. Since we demonstrated that BALF1 accumulated in LC3-positive vesicles, a process that also depends on the putative LIR motif, we propose that BALF1 may compete with other LIR-containing proteins for targeting to the autophagosome membranes, thereby preventing them from being degraded by autophagy. This might be especially important in the case of p62, since it has multiple domains that mediate its interactions with various partners. As such, p62 serves as an essential signaling hub and is involved in autophagy, oxidative stress signaling, and cancer [64]. LC3 and BALF1 were co-purified in cell fractionation experiments. However, both proteins were mainly insoluble under mild extraction conditions and could not be subjected to immunoprecipitation. Stronger detergents and chaotropic agents solubilized both LC3 and BALF1 but were not compatible with immunoprecipitation procedures. Other biochemical approaches are therefore needed to explore putative LC3-BALF1 interaction. Interestingly, subtle variations within the LIR motif and surrounding amino acids have been shown to determine which ATG8 protein can be targeted [49,50]. In that respect, the absence of a detectable interaction between BALF0/1 and LC3B may indicate that BALF0/1 could interact with another ATG8 protein, which is currently under investigation in our laboratory. Since BALF0 and BALF1 are not degraded by autophagy (Figure 3C and Figure 4C), they are more likely to interact with an ATG8 protein that is not subjected to autophagy-mediated degradation, most likely on the outer phagophore membrane. To date, only two viral proteins have been reported to interact directly with LC3 family proteins: human immunodeficiency virus type 1 (HIV-1) viral infectivity factor (Vif) and influenza A virus (IAV) Matrix 2 (M2) [65,66]. M2 is the only one that contains an experimentally validated LIR motif, which is required for the redistribution of LC3 to plasma membrane in IAV-infected cells [66]. Last but not least, modulation of autophagy by BALF0 and BALF1 may directly or indirectly contribute to virion morphogenesis. Although initial work by Johannsen and colleagues did not identify BALF0 and BALF1 in purified EBV virions [67], another study led to co-purification of BALF0 and BALF1 with BSRF1 [68], an EBV tegument protein that is homologous to HSV-1 unique long 51 (UL51) and HCMV UL71, which is involved in virion egress. This result, which shed new light on the putative function of BALF0 and BALF1 during the EBV lytic cycle, is strongly reminiscent of a recent report indicating that vBcl-2 from KSHV could similarly interact with tegument protein ORF55 [69]. However, KSHV vBcl-2 interaction with ORF55 was shown to be critical for the KSHV lytic cycle, whereas BALF0 and BALF1 proteins may be dispensable for virus production, as shown by Altman and co-workers [32]. Although BALF0 and BALF1 proteins may not contribute to virion release, they may affect virion infectivity, which would deserve further investigation. Similarly, genetic studies will be required to precisely delineate BALF0 and BALF1 regions that are required for apoptosis inhibition, autophagy stimulation, and virion morphogenesis, three functions that have recently been described in KSHV vBcl-2 [69].

## Supplementary Materials

The following are available online at https://www.mdpi.com/1999-4915/11/12/1099/s1, Table S1: GenBank accession numbers of sequences used for alignment in this study. Table S2: Primer sequences used in this study. Figure S1: Autophagic modulation by BALF0. Figure S2: Colocalization between BALF0 and GFP-LC3 vesicles.
