# Supplementary material for: Epstein–Barr Virus BALF0 and BALF1 Modulate Autophagy
Source: Viruses. 2019 Nov 27;11(12):1099. doi: 10.3390/v11121099 (PMC6950364; doi:10.3390/v11121099)
Supplement: Supplementary file 1 [file viruses-11-01099-s001.zip › Supplementary File/Figure S2.docx]

**Figure S2**


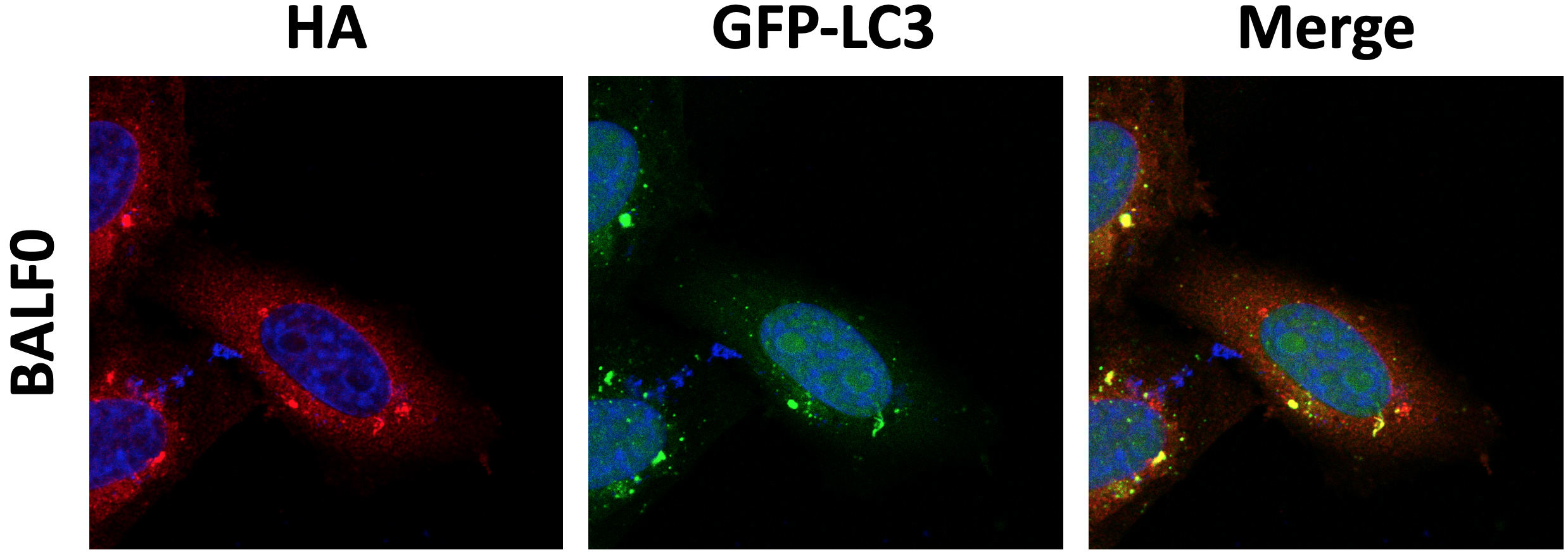


**Figure S2.** Colocalization between BALF0 and GFP-LC3 vesicles. Representative images of HeLa cells stably expressing GFP-LC3 following transfection with a BALF0-HA encoding plasmid. The cells were observed 24h post-transfection by confocal microscopy. BALF0 was detected by immunofluorescence with an anti-HA antibody (red). Nuclei were stained with Hoechst 33342 (blue). Fluorescence intensities of BALF0-expressing cells have been modified for visibility.
