# Supplementary material for: Epstein–Barr Virus BALF0 and BALF1 Modulate Autophagy
Source: Viruses. 2019 Nov 27;11(12):1099. doi: 10.3390/v11121099 (PMC6950364; doi:10.3390/v11121099)
Supplement: Supplementary file 1 [file viruses-11-01099-s001.zip › Supplementary File/Table S2.docx]

Table S2. Primer sequences used in this study

| Program | Target | Forward primer 5’-3’ | Reverse primer 5’-3’ |
| --- | --- | --- | --- |
| qRT-PCR | BALF0/1 | GAAACTACCTGGATGACCACAA | CAAACCAGAGTCTGCGATAGAG |
|  | Cyclophilin | GCCTTAGCTACAGGAGAGAA | TTTCCTCCTGTGCCATCTC |
| Plasmid | BALF0/1 | AGTCCAGTGTGGTGGAGCGATGAACCTGGCCATTGCT | GATATCTGCAGAATTTTAAGCGTAATCTGGAACATCGTATGGGTACAAAGATTTCAGGAAGTC |
| Mutagenesis | BALF0 | GCCTGACGAGACCGGTAGGCCAGCCAAGTC | GACTTGGCTGGCCTACCGGTCTCGTCAGGC |
|  | BALF1 | GTGGTGGAGCGATATCCCTGGCCATTGCTC | GAGCAATGGCCAGGGATATCGCTCCACCAC |
|  | W146A | CACTACGACTACGCTAGCCGGCTCAGGGTG | CACCCTGAGCCGGCTAGCGTAGTCGTAGTG |
|  | W146A-L149A | GACTACGCTAGCCGGGCGCGCGTGGTGCTGTGCTAC | GTAGCACAGCACCACGCGCGCCCGGCTAGCGTAGTC |
