# Supplementary material for: Epstein–Barr Virus BALF0 and BALF1 Modulate Autophagy
Source: Viruses. 2019 Nov 27;11(12):1099. doi: 10.3390/v11121099 (PMC6950364; doi:10.3390/v11121099)
Supplement: Supplementary file 1 [file viruses-11-01099-s001.zip › Supplementary File/Table S1.docx]

Table S1. GenBank accession numbers of sequences used for alignment in this study

| Group | Name | Organism | Accession number |
| --- | --- | --- | --- |
| Human Bcl-2 homolog | Bak | Homo sapiens | NP_001179 |
|  | Bax | Homo sapiens | NP_620116 |
|  | Bcl-2 | Homo sapiens | NP_000624 |
|  | Bcl-x_L_ | Homo sapiens | CAA80661 |
| Viral Bcl-2 homolog | BHRF1 | Human gammaherpesvirus 4 | YP_401646 |
|  | Ks-Bcl-2 | Human gammaherpesvirus 8 | YP_001129368 |
|  | vBcl-2 | Saimiriine gammaherpesvirus 2 | CAC84311 |
| BALF1 homolog | BALF0 | Human gammaherpesvirus 4 | CAA24810 |
|  | BALF1 | Human gammaherpesvirus 4 | YP_401718 |
|  | BALF1 | Gorilline gammaherpesvirus 1 | AAK60342 |
|  | BALF1 | Macaca arctoides gammaherpesvirus 1 | AYA49869 |
|  | BALF1 | Macacine herpesvirus 4 | YP_068013 |
|  | BALF1 | Panine gammaherpesvirus 1 | AAK01917 |
|  | BALF1 | Papiine gammaherpesvirus 1 | AAK01916 |
|  | ORF1 | Callitrichine gammaherpesvirus 3 | NP_733853 |
|  | BALF1 | Alcelaphine gammaherpesvirus 1 | NP_597933 |
|  | BALF1 | Dolphin gammaherpesvirus 1 | YP_009388510 |
|  | BALF1 | Equid gammaherpesvirus 2 | NP_042601 |
|  | BALF1 | Equid gammaherpesvirus 5 | YP_009118395 |
|  | BALF1 | Myotis gammaherpesvirus 8 | YP_009229842 |
|  | Bcl-2 | Rhinolophus gammaherpesvirus 1 | YP_009551815 |
|  | orf4 | Alcelaphine gammaherpesvirus 2 | YP_009044392 |
|  | Ov4.5 | Ovine gammaherpesvirus 2 | YP_438132 |
|  | v-bcl2 | Porcine lymphotropic herpesvirus 1 | YP_009505335 |
|  | v-bcl2 | Porcine lymphotropic herpesvirus 2 | YP_009505382 |
|  | v-bcl2 | Porcine lymphotropic herpesvirus 3 | AAO12311 |
|  | vBcl-2 | Bovine gammaherpesvirus 6 | YP_009041986 |
|  | vbcl-2b | Phascolarctid gammaherpesvirus 1 | AZB49183 |
