# Supplementary material for: Epstein–Barr Virus BALF0 and BALF1 Modulate Autophagy
Source: Viruses. 2019 Nov 27;11(12):1099. doi: 10.3390/v11121099 (PMC6950364; doi:10.3390/v11121099)
Supplement: Supplementary file 1 [file viruses-11-01099-s001.zip › Supplementary File/Figure S1.docx]

**Figure S1**


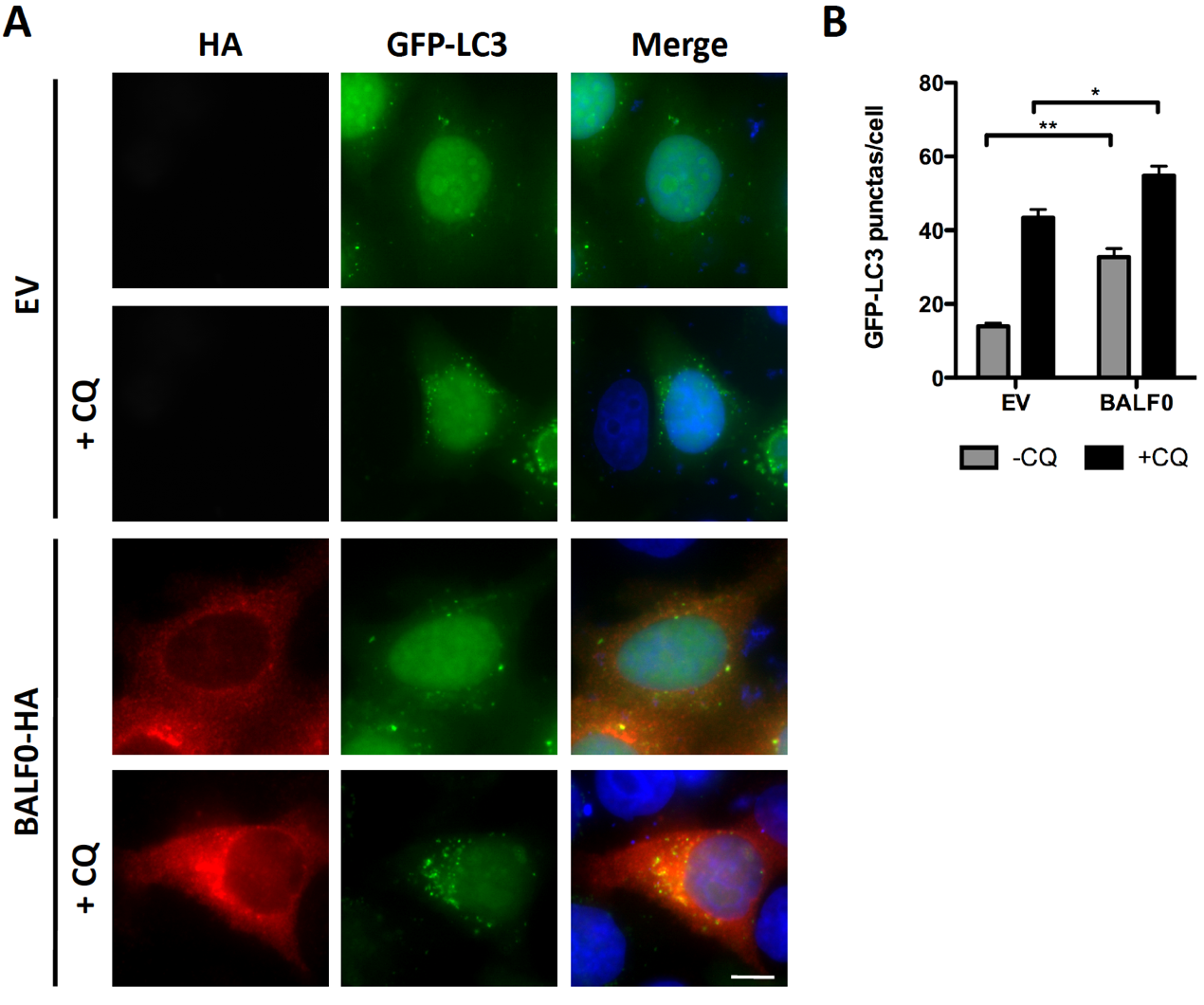


**Figure S1.** Autophagic modulation by BALF0. (A) Representative images of GFP-LC3 HeLa cells transfected with empty vector (EV) or BALF0-HA encoding plasmid (24h post-transfection) in the presence of absence of chloroquine (CQ). BALF0-transfected cells were visualized by immunofluorescence with an anti-HA antibody (red). Nuclei were stained with Hoechst 33342 (blue). Scale bars = 20μm. Fluorescence intensities of BALF0-expressing cells have been modified for visibility. (B) Autophagosomes formation was evaluated by quantifying the number of GFP-LC3 puncta per cell following transfection. The results are the mean± SEM of three independent experiments, and 50 cells were analyzed per assay. **P<0.01; *P<0.05.
